# Thyroid-Stimulatory Antibody as a Predictive Factor for Graves’ Disease Relapse

**DOI:** 10.7759/cureus.22190

**Published:** 2022-02-14

**Authors:** Tiago Da Silva Santos, José Carlos Oliveira, Cláudia Freitas, André Couto de Carvalho

**Affiliations:** 1 Division of Endocrinology, Diabetes and Metabolism, Centro Hospitalar e Universitário do Porto, Porto, PRT; 2 Division of Clinical Pathology, Centro Hospitalar e Universitário do Porto, Porto, PRT

**Keywords:** thyroid pathologies, relapse, thyroid stimulatory antibody, thyrotropin receptor antibody, graves´disease

## Abstract

Introduction: Thyroid-stimulatory antibody (TSAb) assays have been recently optimized, potentially allowing to determine thyrotropin receptor antibodies' (TRAbs) functionality in routine clinical practice. We aimed to determine TSAb's predictive role of relapse at antithyroid drug (ATD) withdrawal in Graves’ disease (GD).

Methods: Retrospective study of GD patients with stable normal thyroid function under low ATD doses that were proposed for withdrawal. Thyroid function tests and TRAb and TSAb levels were obtained at ATD suspension and every three to six months after that, for a minimum of 16 months. Clinical factors associated with GD relapse, such as age at diagnosis, sex, smoking status, thyroid volume, and presence of orbitopathy, were also evaluated.

Results: Thirty-five patients with GD were included for analysis, with a median follow-up period of 24 months, during which 14 patients (40%) relapsed. Relapse was more common in patients with positive TSAb than patients with negative TSAb at ATD withdrawal (79% vs. 33%, p=0.01). Relapse-free survival was shorter in TSAb-positive patients (p=0.01). There were no differences in relapse rates according to TRAb positivity at ATD withdrawal (42.9% vs. 36.4%, p=0.74). We also did not find any differences in relapse rate regarding age, sex, smoking status, thyroid volume, or presence of Graves’ orbitopathy. On multivariate analysis, only TSAb positivity at ATD withdrawal was independently associated with relapse (hazard ratio [HR] 6.63, 95% confidence interval [CI], 1.30-33.7, p=0.02).

Conclusion: At ATD withdrawal, TSAb-positive patients demonstrated a higher risk for GD relapse. Measuring TSAb before ATD suspension, instead of TRAbs, could become an important tool for the clinical management of these patients.

## Introduction

Graves’ disease (GD) is an autoimmune disease that represents the most common cause of hyperthyroidism in iodine-replete areas, with a lifetime risk around 3% in women and 0.5% in men [1,2]. Both genetic and environmental factors, such as stressful life events, high iodine intake, and smoking, predispose to GD [3,4]. One of the hallmarks associated with GD is the production of autoantibodies to well-defined thyroidal antigens, such as thyrotropin receptor antibodies (TRAbs), subsequently inducing the production and release of thyroid hormone, the proliferation of the thyrocytes, and further enlargement of the thyroid gland [5]. These autoantibodies are characterized for being functionally significant, given that they can either act as agonists that stimulate thyroid growth (stimulating immunoglobulins), antagonists that block the activity of thyroid-stimulating hormone (TSH) (blocking immunoglobulins), or being functionally inactive (neutral immunoglobulins). TRAbs and more specifically thyroid-stimulatory antibodies (TSAbs) are the causative agent of GD in patients suspected of having hyperthyroidism, constituting a specific biomarker of GD [5-7].

Antithyroid drugs (ATDs) are an effective therapeutic modality for the treatment of GD patients, given their capacity to reduce thyroid hormone production and their putative immunosuppressive effect [1]. However, hyperthyroidism relapse rate after ATD withdrawal remains high, ranging from 30% to 70% over the literature [3]. It would be undoubtedly useful to predict the course and relapse risk of GD during follow-up, allowing an early shift to a definite treatment option if the risk of relapse could be easily determined. Clinical factors such as goiter size, thyroid hormone levels, smoking habits, and TRAbs' values at baseline have been identified as important risk factors for relapse, while the impact of gender, young age, and orbitopathy remains uncertain [4,8].

The conventional TRAb immunoassays measure not only thyroid-stimulating immunoglobulin but also thyroid-blocking immunoglobulins and neutral immunoglobulins [9,10]. These assays are only able to report the presence or absence of TRAbs and their concentration levels but cannot indicate their overall functional predominance [11,12]. In contrast, cell-based bioassays and more recent sensitive quantitative blood immunoassays are able to distinguish TRAbs' functionality, specifically detecting TSAb and therefore allowing differentiation between the thyrotropin receptor antibodies' subtypes (blocking/neutral/stimulating) [12-15].

This study aimed to assess the predictive value of TSAb levels in hyperthyroidism relapse after ATD withdrawal in GD patients.

## Materials and methods

Patients

This retrospective observational study enrolled 117 GD patients treated with ATDs that achieved stable normal thyroid function levels under low ATD dose and were proposed for withdrawal between 2016 and 2020.

GD diagnosis was based on the following criteria: serum TSH levels below lower limit of normality (<0.3 µIU/mL) with high free thyroxine (FT4 >1.58 ng/dL) and/or high free triiodothyronine (FT3 >4.36 pg/mL) associated with increased serum TRAb levels (TRAb levels >1.75 IU/L or TSAb levels >0.55 IU/L) and the presence of appropriate clinical features such as symptoms of hyperthyroidism, presence of a diffuse goiter (defined by ultrasonography), or thyroid orbitopathy.

**Figure 1:**
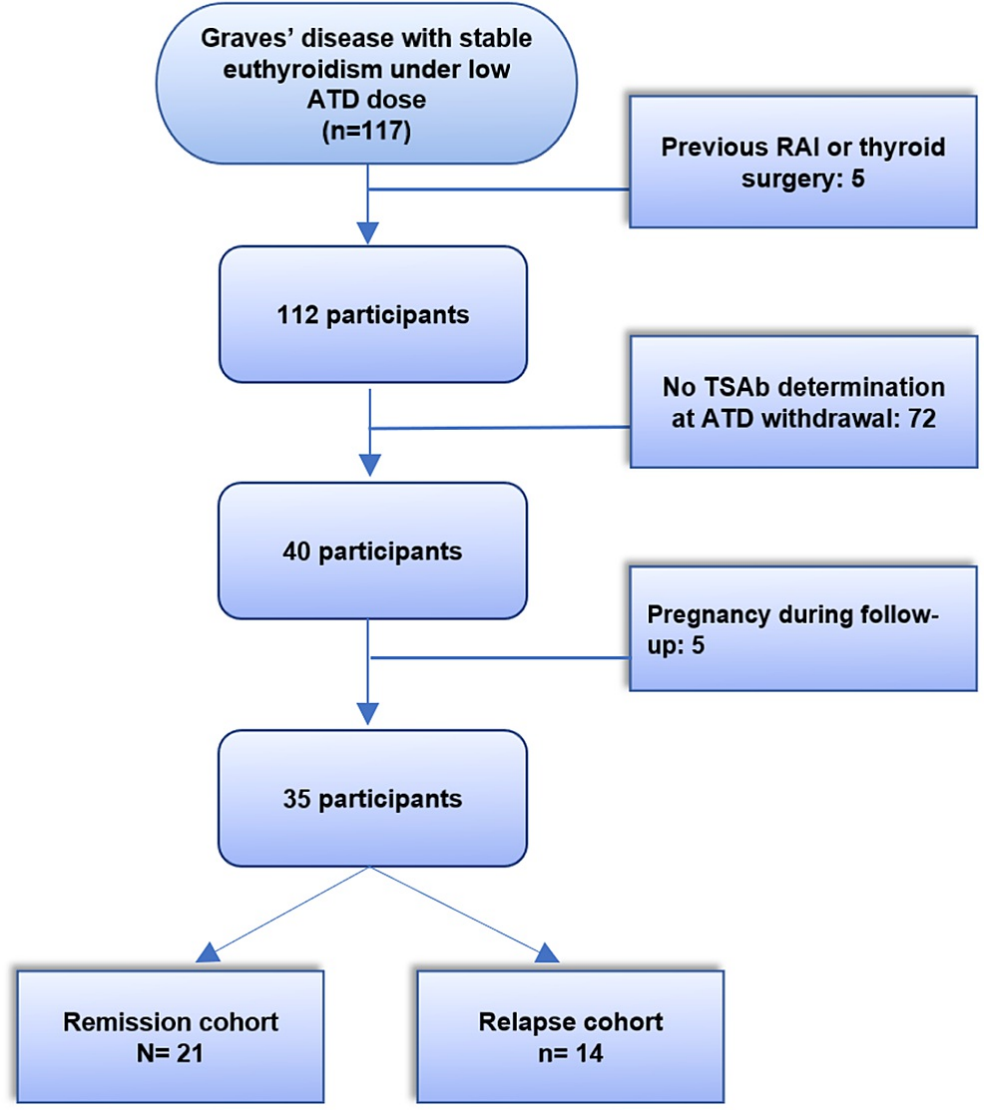
Patient selection flowchart ATD, antithyroid drugs; RAI, radioactive iodine; TSAb, thyroid-stimulating antibody

Patients without any TSAb level determination within the study period were excluded from the analysis, as well as patients under 18 years old or who became pregnant during the follow-up period. Individuals with previous radioactive iodine therapy or thyroid surgery were also excluded (Figure 1).

ATD treatment and follow-up examinations were performed under a uniform protocol using a dose-titrating regime, mostly with methimazole (MMI) throughout the study period. ATDs were suspended when the serum FT4 and TSH levels of the patient became within the normal range for at least six months under the minimum maintenance dose of ATDs (MMI ≤2.5 mg/day and propylthiouracil ≤50 mg/day). After ATD therapy’s suspension, thyroid function tests and TRAb plus TSAb levels were obtained every three to six months for at least 16 months.

Remission was defined as a state of euthyroidism (demonstrated by normal ranges of FT3, FT4, and TSH levels) following the withdrawal of ATD for more than six months. Relapse was defined if hyperthyroidism (FT4 or FT3 above the higher limit of normality and TSH levels below the lower limit of normality) was detected at any time during follow-up. TSAb and TRAb levels as well as clinical factors commonly associated with GD relapse, such as age at diagnosis, sex, smoking status, thyroid volume, and presence of ophthalmopathy, were investigated.

Laboratory measurements

Serum levels of TSH, FT3, and FT4 were measured with the Cobas e601 analyzer (Elecsys, Roche Diagnostics, Mannheim, Germany) according to the manufacturer’s instructions. Reference ranges for TSH, FT4, and FT3 were of 0.30-3.94 µIU/mL, 0.95-1.57 ng/dL, and 2.42-4.36 pg/mL, respectively. TRAb was measured with a competitive Electro Chem Luminiscens Immunoassay (Elecsys Anti-TSHR) according to the manufacturer’s instructions (Roche Diagnostics, Mannheim, Germany). The reportable range for TRAb was 0.3-40.0 IU/L and levels ≥1.75 IU/L were considered positive, with a sensitivity of 96% and specificity of 99%. Inter- and intra-assay variability was determined between 2.4-28.8% and 1.4-14.9%, respectively. Repeatability coefficient of variation (CV) and within-laboratory CVs were ≤7.5% and ≤9.1% across the measuring range, respectively. This method was performed according to the WHO's first International Standard (IS) for measuring TRAb, NIBSC code 90/672. TSAb concentrations were measured with an automated chemiluminescent bridge immunoassay (IMMULITE 2000 TSI assay, Siemens Healthcare Diagnostics, Tarrytown, NY, USA) according to the manufacturer’s instructions. The reportable range of TSAb was 0.10-40.0 IU/L and levels ≥0.55 IU/L were considered positive, with a sensitivity of 100% and specificity of 99%. Repeatability CV and within-laboratory CVs were ≤7.0% and ≤8.3% across the measuring range, respectively. The WHO's second IS for thyroid-stimulating antibodies, NIBSC code 08/204, was used to calibrate this method.

Statistical analysis

Continuous and categorical variables are presented as mean ± standard deviation (SD) or medians with interquartile ranges (IQR) and numbers with proportions, respectively. For continuous quantitative variables, distribution normality was tested through histogram observation and Kolmogorov-Smirnov test analysis. The student's t-test and the Mann-Whitney U test were used to compare continuous variables with normal and non-normal distribution between groups, respectively. Pearson's chi-square test was used to compare categorical data. The hazard ratio (HR) and 95% confidence interval (CI) used to evaluate the association between TSAb levels at withdrawal and relapse were derived using Cox's proportional hazards modeling. All analyses were further evaluated by stratifying for potential confounder factors which were selected based on previous literature and biological plausibility, such as age, sex, smoking status, presence of goiter or ophthalmopathy, TRAb levels, and duration of ATD treatment. Relapse-free survival (RFS) rates were calculated using the Kaplan-Meier method and compared to the log-rank test according to the TSAb levels. All statistical tests were two-tailed and performed using statistical software (SPSS v.25.0 for Windows; IBM Co, Armonk, NY). A p-value of <0.05 was considered statistically significant.

## Results

From a total of 117 patients with GD, 35 patients were included for the analysis accordingly to our inclusion criteria (Figure 1). Their baseline characteristics are listed in Table 1. The mean age at GD diagnosis was of 45.5 ± 14.8 years, 29 patients (83%) were female, and seven (20%) were active smokers. Eight patients (23%) had a visible goiter and six (17%) presented Graves’ orbitopathy at diagnosis. Thirty-one (89%) were treated with MMI for a median treatment duration of 18 months (IQR, 12.0-24.0) and a median duration of euthyroid status under a minimal ATD maintenance dose of 12 months (6.0-14.0). After a median follow-up period of 24.0 months (IQR, 21.0-26.0), 14 patients (40%) observed relapse hyperthyroidism (Table 1).

**Table 1:** Baseline characteristics of patients included Continuous variables are presented as mean ± SD  or median (interquartile range) while categorical variables are expressed as n (%). ATD, antithyroid drug; FT3, free triiodothyronine; FT4, free thyroxine; NA, not applicable; TRAb, thyrotropin receptor binding antibody; TSAb, thyroid-stimulatory antibody  levels; TSH, thyroid-stimulating hormone.

Patient data	Total (n=35)	Remission group (n=21)	Relapse group (n=14)	p-Value	
Age at diagnosis, years	45.5±14.8	45.4±15.1	45.5±14.8	0.98	
Female gender, n (%)	29 (83)	17 (81)	12 (86)	0.71	
Active smokers, n (%)	29 (83)	17 (81)	12 (86)	0.71	
Presence of goiter, n (%)	8 (23)	5 (24)	3 (21)	0.59	
Small (<40 g)	5 (62)	2 (40)	3 (100)	0.18	
Medium-large (>40 g)	3 (38)	3 (60)	0	NA	
Graves’ orbitopathy, n (%)	6 (17)	4 (19)	2 (14)	0.54	
Thyroid function parameters at diagnosis					
TSH (µIU/L)	0.008 (0.005-0.01)	0.01 (0.005-0.01)	0.005 (0.005-0.008)	0.19	
FT4 (ng/dL)	2.25 (1.60-3.28)	2.19 (1.38-3.12)	2.40 (1.87-4.79)	0.19	
FT3 (pg/mL)	7.60 (3.94-11.60)	5.20 (3.89-11.07)	8.21 (5.74-14.55)	0.30	
FT3/FT4 ratio	3.06 (2.30-3.42)	3.06 (2.57-3.26)	3.19 (2.26-3.50)	0.88	
Thyroid function parameters at ATD withdrawal					
TSH (µIU/L)	1.98 (1.10-3.45)	2.39 (1.10-4.16)	1.39 (1.26-3.32)	0.70	
FT4 (ng/dL)	1.07 (0.94-1.22)	1.07 (0.98-1.21)	1.05 (0.84-1.26)	0.50	
FT3 (pg/mL)	2.97 (2.65-3.20)	2.99 (2.63-3.28)	2.84 (2.68-3.19)	0.74	
FT3/FT4 ratio	2.80 (2.34-3.04)	2.74 (2.31-2.93)	2.82(2.36-3.31)	0.65	
Positivity of TRAb, n (%)	12 (34)	7 (33)	5 (36)	0.58	
TRAb levels (IU/L)	1.03 (0.76-2.07)	0.92 (0.70-2.02)	1.42 (0.86-2.75)	0.54	
Positivity of TSAb, n (%)	18 (51)	7 (33)	11 (79)	0.010*	
TSAb levels (IU/L)	0.52 (0.19-2.60)	0.28 (0.10-1.38)	1.41 (0.60-2.77)	0.018*	
Methimazole, n (%)	31 (89)	20 (90)	12 (86)	0.63	
Propylthiouracil, n (%)	4 (11)	2 (10)	2 (14)	0.53	
Treatment duration of ATD, months	18.0 (12.0-24.0)	17.0 (12.0-28.0)	21.0 (18.0-24.0)	0.48	
Euthyroid duration under ATD, months	12.0 (6.0-14.0)	12.0 (6.0-14.0)	11.5 (7.0-16.0)	0.77	
Follow-up duration, months	24.0 (21.0-26.0)	24.0 (20.0-26.0)	24.0 (22.0-26.0)	0.36	

There were significantly more cases of relapse in TSAb-positive patients than in TSAb-negative patients (79% vs 33%, p=0.01). Patients with GD relapse demonstrated significantly higher TSAb levels at ATD discontinuation (1.41 vs 0.28 IU/L, p=0.018) (Table 1). Our sample showed no differences in the relapse rate according to TRAb positivity at ATD withdrawal (36% vs 33%, p=0.48). Within the relapse group, 6 (55%) patients were TSAb-positive but TRAb-negative at ATD withdrawal. We did not find any significant differences in the relapse rate regarding age, sex, smoking status, thyroid volume, presence of Graves’ orbitopathy, treatment duration with ATDs, duration of euthyroid status, and thyroid function parameters at diagnosis or ATDs withdrawal. 

**Figure 2:**
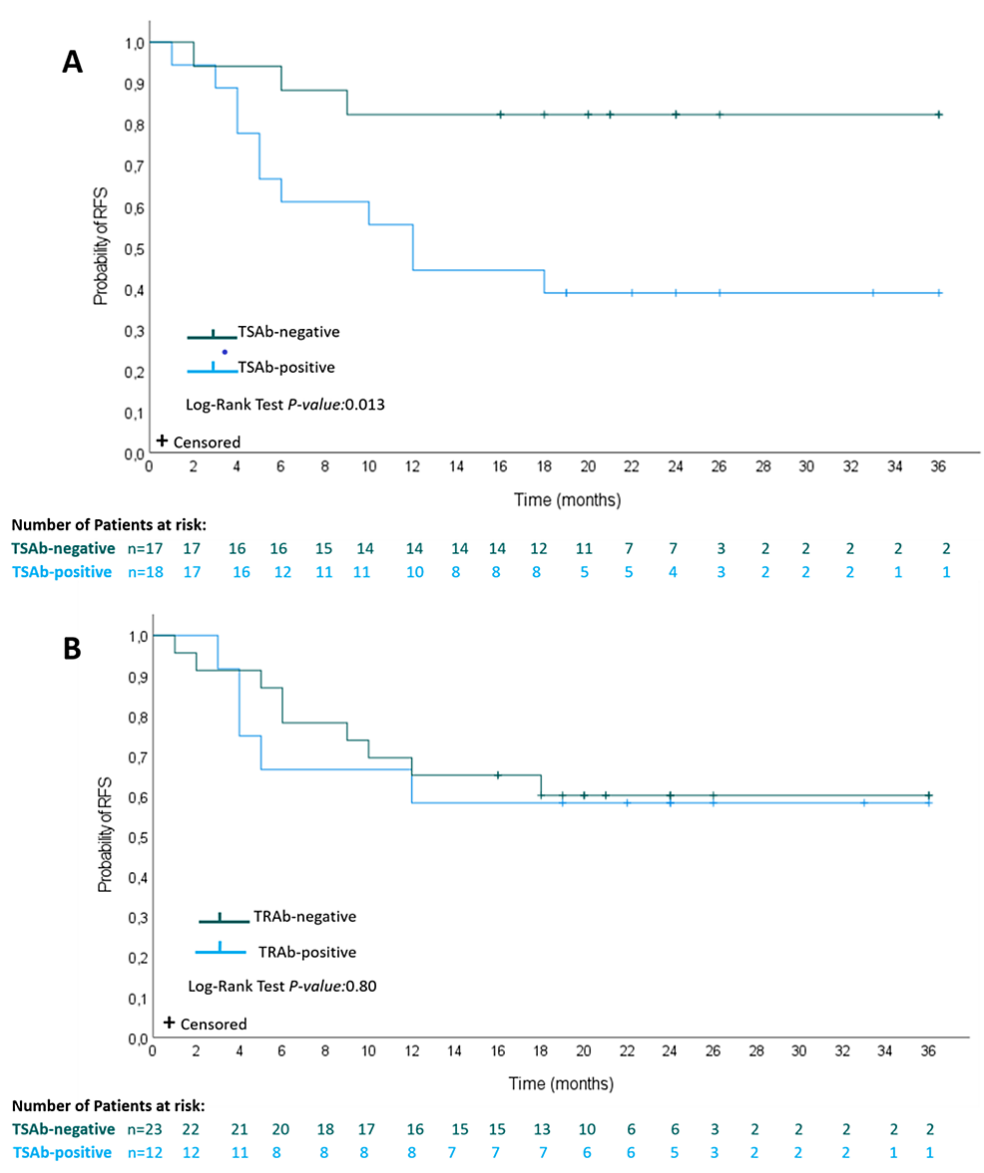
Kaplan-Meier plots of relapse-free survival of patients after antithyroid drugs withdrawal according to TSAb levels (A) and TRAb levels (B) p-Values were based on an unstratified log-rank test. CI, 95% confidence interval; HR, hazard ratio; TRAb, thyrotropin receptor binding antibody; TSAb, thyroid-stimulatory antibody  levels.

TSAb-positive patients demonstrated shorter RFS than TSAb-negative patients (p=0.013) (Figure 2). In contrast, there was no significant difference in RFS between patients with positive and negative TRAb levels (p=0.80) (Figure 2).

On univariate analysis, the TSAb level at ATD withdrawal was the only significant factor associated with relapse, (HR, 4.37; 95% CI, 1.22-15.71; p=0.024). TSAb positivity at ATD withdrawal was independently associated with relapse both on a multivariate analysis model adjusted only for TRAb status (HR, 6.43; 95% CI, 1.63-25.32; p=0.008) and on a multivariate analysis model adjusted for multiple known factors commonly associated with GD relapse (age at diagnosis, sex, treatment duration under ATDs, smoking status, presence of goiter or thyroid-associated orbitopathy) with a six-fold increased risk for relapse (HR, 6.63; 95% CI, 1.30-33.73; p=0.02) (Table 2).

**Table 2:** Cox's proportional hazard modeling for predicting Graves’ disease relapse Cox's proportional hazard modeling: multivariate analysis included  the following covariates: age at diagnosis, sex, treatment duration of ATDs, presence of goiter or thyroid-associated orbitopathy, smoking status, and positive TRAb and TSAb levels at ATD withdrawal. ^a^TRAb positivity was defined as TRAb levels  >1.75 IU/L. ^b^TSAb positivity was defined as TSAb levels >0.55 IU/L. ATD, antithyroid drug; CI, confidence interval; HR, hazard ratio; TRAb, thyrotropin-binding antibody; TSAb, thyroid-stimulatory antibody.

Parameter	Univariate			Multivariate		
	HR	95% CI	p-Value	HR	95% CI	p-Value
Age at diagnosis (<45 years )	0.84	0.29-2.42	0.75	0.87	0.23-3.30	0.83
Sex (female)	1.07	0.23-4.78	0.93	0.34	0.05-2.27	0.26
Treatment duration of ATDs (<18 months)	0.42	0.12-1.50	0.18	1.80	0.43-7.60	0.42
Goiter (medium to large)	0.89	0.26-3.30	0.86	0.74	0.17-3.26	0.69
Thyroid-associated orbitopathy (yes)	0.74	0.18-3.60	0.69	0.29	0.42-1.98	0.21
Smoking (yes)	1.73	0.50-5.10	0.35	2.53	0.53-12.02	0.24
Positive TRAb level at ATD withdrawal^a^	1.29	0.34-3.86	0.65	0.42	0.10-1.68	0.22
Positive TSAb level at ATD withdrawal^b^	4.37	1.22-15.71	0.024*	6.63	1.30-33.73	0.023*

## Discussion

In our work, GD patients with persistent TSAb-positive levels demonstrated a six-fold increased risk for relapse after ATD withdrawal. This observation was independent of the overall TRAb status.

Treatment options in GD after a course of ATD remain a challenge for individual patient management. Currently, there is no consensual index for identifying which patients are better to respond to and obtain remission after ATD therapy. Due to its active pathogenic role, TRAb measurement at the time of ATD withdrawal has been used as a predictor of GD relapse but published data are inconsistent [16,17].

TSAb is a thyrotropin receptor autoantibody subtype found in GD patients that is able to bind to thyrotropin receptors and stimulate thyroid cell proliferation and function [5]. Currently, two different types of methods for assessing TSAb are available. The first method uses Chinese hamster ovary cells transfected with the cloned TSH receptor cDNA, being highly sensitive [11]. These cell-based bioassays can distinguish between different TRAb subtypes through their effect on cyclic adenosine monophosphate (cAMP) production [11]. The TSAb-Mc4 bioassay is one of the most used and was bio-engineered to constitutively express a chimeric TSH receptor and a cAMP-inducible luciferase reporter gene, enabling TSAb quantification with improved accuracy and sensitivity when compared to previous wild-type TSH receptor bioassays [18,19]. The second method includes a bridge immunoassay design to measure TSAb serum levels based on a distinctive technology and assay format that is available in an automated commercial platform (IMMULITE 2000). This method employs a pair of recombinant human-TSH-R constructs in a sandwich format, constituted by capture and signal chimeric receptors which allow direct detection and measurement of thyroid-stimulating autoantibodies to TSH receptors [11]. As a common point, both methods allow TRAb functional activity assessment and present a major difference from conventional TRAb-binding immunoassays [9,10,12].

TSAb levels have been associated with the active phase as well as GD relapse with better remission rates when found to be negative/mildly elevated [20,21]. Thereby, TSAb levels measurement at the time of drug withdrawal has been proposed to be a good predictive factor for long-term remission in GD [21-24]. Over the literature, however, it remains controversial which TRAb assay is more useful for this role. The first meta-analysis evaluating TRAb’s value in predicting GD relapse was published more than 25 years ago with 10 studies analyzed (five prospective and five retrospective studies) and demonstrated that both a decline in TRAb levels during treatment as well as negative TRAb level at the end of treatment were both predictive of long-term remission [23]. More recently, several studies have evaluated the value of current TRAb assays for predicting GD relapse with some conflicting results [17,20,25,26]. Capelli et al. correlated TRAb levels with long-term remission in a prospective design study and found that median TRAb levels at ATD withdrawal were significantly higher in the relapse group [20]. On the other hand, Quadbeck et al. failed to demonstrate any difference in RFS between patients with positive versus negative TRAb levels at ATD withdrawal [27]. It remains unclear why TRAb can be such an excellent tool for the diagnosis of GD but a rather poor performer in the prediction of relapse. 

On the other hand, some new studies have demonstrated that TSAb levels outperform TRAb assays in predicting GD relapse after ATD withdrawal [16,22,23]. Giuliani et al. showed in a five-year prospective study using a TSAb bioassay (Thyretain Mc4 assay) a trend toward better relapse predictive value and sensitivity when compared to TRAb immunoassays [16]. Another study, by Kwon et al., reported that TSAb-positive patients demonstrated higher RFS levels than TSAb-negative patients while failing to find such an association between patients with positive and negative TRAb levels [24]. Finally, a prospective study published in 2020 by Kahaly et al. showed that serum TSAb levels do mirror synchronous GD severity. The authors have found that, when compared to TRAb, TSAb levels were a better early predictor of disease progression, remission, or response to therapy and that TSAb positivity independently predicted GD relapse after adjustment for other prognostic variables. Specifically, within this study, the predictive value of the TSAb was evaluated at baseline before treatment under ATD [28]. Similarly, in our work, patients with positive TSAb demonstrated shorter RFS rates than patients with negative TSAb (HR, 4.37; 95% CI, 1.22-15.71; p=0.013), while we did not find such a difference when evaluating TRAb status (HR, 1.29; 95% CI, 0.10-2.25; p=0.80).

Full evidence that IMMULITE 2000® assay is fully specific for TSAb is lacking. Diana et al. suggested that this immunoassay cannot fully differentiate between TSAb and TSH-R blocking antibodies as bioassays can [14]. On the other hand, a recent study by Hu et al. reinforces that this TSAb immunoassay provides higher positive predictive values than the corresponding TRAb assays [29]. Despite some disagreement among published work, our data, with small sample size, cannot fully validate this assumption and demonstrate that this TSAb immunoassay could be a better predictor of GD relapse after ATD withdrawal than standard TRAb assays.

Multiple factors, including younger age, male sex, smoking, presence of goiter or Graves’ orbitopathy, smoking status, and higher thyroid hormone levels, have been commonly associated with a higher risk of relapse [7,8,30]. However, we were unable to demonstrate these associations in our study. This finding was probably linked to the small number of patients included, which may reduce the statistic power for finding less strong associations.

This study has several limitations which could also influence our results. First, its retrospective design should be acknowledged with some selection bias associated. Within our initial cohort of 117 GD patients, 72 patients were excluded for lacking any TSAb determination at ATD withdrawal, which significantly shortened our sample's size. This study was also performed in an iodine-insufficient geographical, and, therefore, our results should not be generalized to other populations with distinctive iodine repletion status [5]. Finally, the median follow-up period was 24 months, which may have been relatively short for evaluating long-term remission status, although most GD recurrences tend to occur within the first 12 months after ATD withdrawal [5].

As strengths of our work, we emphasize the relatively clinical homogeneity of this patients’ sample and their clinical management, as well as the simultaneous TRAb and TSAb determination, which allowed direct comparison between these two assays. Finally, we also present one of the first studies that evaluate commercially available TSAb immunoassays’ predictive value on GD relapse, presenting an easy-to-use tool with a potentially important role in routine clinical practice.

## Conclusions

In the current study, we assessed the predictive value of TSAb levels in hyperthyroidism relapse after ATD withdrawal in GD patients. Our results showed that GD patients with persistent TSAb-positive levels demonstrated a six-fold increased risk for relapse after ATD withdrawal, independent of their overall TRAb status. We have found that TSAb levels are better predictors of GD relapse after ATD withdrawal than TRAbs. Measuring TSAb under stable low ATD doses could become an important tool for ATD management in GD patients.
